# Puffer Fish Products in the United States

**DOI:** 10.14252/foodsafetyfscj.D-25-00012

**Published:** 2025-12-19

**Authors:** Jonathan R. Deeds, Sara C. McGrath, Sara M. Handy, Karen A. Swajian

**Affiliations:** 1 Office of Laboratory Operations and Applied Science, Human Foods Program, U.S. Food and Drug Administration,5001 Campus Drive, College Park, Maryland 20740, USA; 2 Office of Food Chemical Safety, Dietary Supplements, and Innovation, Human Foods Program, U.S. Food and Drug Administration, 5001 Campus Drive, College Park, Maryland 20740, USA; 3 Office of Microbiological Food Safety, Human Food Program, U.S. Food and Drug Administration, 5001 Campus Drive, College Park, Maryland 20740, USA

**Keywords:** puffer fish, Puffer Fish Poisoning, tetrodotoxin, saxitoxin, *Lagocephalus lunaris*, *Sphoeroides nephelus*.

## Abstract

Puffer fish and products containing puffer fish are highly regulated and restricted in the United States due to the potential presence of the alkaloid toxins tetrodotoxins (TTX) and saxitoxins (STX). Imported and domestic puffer fish are regulated under the U.S. Code of Federal Regulations (21 CFR Part 123 - Fish and Fishery Products) which identifies Hazard Analysis Critical Control Point (HACCP) processes for the control of specific hazards including natural toxins. Additional restrictions are placed on puffer fish depending on the source. The only approved source of imported puffer fish is allowed through an Exchange of Letters between the U.S. Food and Drug Administration and the Japanese Ministry of Health, Labour, and Welfare restricting imported products to the meat, skin, and testicles of *Takifugu rubripes*. Additional restrictions are placed on domestic puffer fish through specific state bans. Despite these efforts, puffer fish poisoning cases still occasionally occur. Illnesses from imported products have mainly been due to TTX in the meat of illegally imported *Lagocephalus lunaris,* while illnesses from domestically sourced products have been due to STX in the meat of *Sphoeroides nephelus* harvested from the Atlantic coast of Florida.

## Introduction

Due to the potential presence of the alkaloid toxins tetrodotoxins (TTX) and saxitoxins (STX), puffer fish are a highly regulated and restricted seafood commodity in the United States. Worldwide, poisoning from consumption of members of the family Tetraodontidae (puffer fish), mainly from TTX – i.e., puffer fish poisoning (PFP) – is one of the most dangerous intoxications from marine species. If a sufficient dose is consumed (estimated at 2–3 mg) death can occur from respiratory failure, typically within 4 to 6 hours with a known range of 20 minutes to 8 hours^1^^,^^2^^)^. STX, produced by several harmful algal species and responsible for paralytic shellfish poisoning (PSP), has also seen found in some puffer fish species^3^^,^^4^^)^. The symptoms of PFP are similar to PSP and traditional bioactivity-based methods of analysis, such as the mouse bioassay and receptor binding assay, cannot easily distinguish these two toxin groups^5^^)^. Therefore, it is possible that some past reports of PFP confirmed using these methods could have been due to STX in addition to TTX.

TTX and STX both act on the central and peripheral nervous systems. The first symptom of intoxication is a slight numbness of the lips and tongue, with an incubation period between 20 minutes to 3 hours after ingestion, depending on the dose. With higher exposures, symptoms can start within minutes. After an initial period of oral numbness, increasing paresthesia in the face and extremities may occur, with sensations of lightness or floating reported in some cases. Headache, epigastric pain, nausea, diarrhea, and/or vomiting may also occur with difficulty in walking also reported. The second stage of intoxication involves progressive paralysis with some victims unable to sit or move. There is increasing respiratory distress, speech is affected, and the victim typically exhibits dyspnea, cyanosis, and hypotension. In high dose exposures, paralysis progresses and convulsions, mental impairment, and cardiac arrhythmia may occur. The victim, although completely paralyzed, may be conscious and, in some cases, completely lucid until shortly before death. There is no antidote for TTX or STX and treatment of PFP is symptomatic and supportive. In most cases, patients who receive ventilatory support have a high expectation for survival^1^^)^. It is known that TTX and STX are depurated from the human body relatively quickly (in days) through the urine and it is generally considered that if victims survive the initial 24 hours, they are expected to recover fully^6^^,^^7^^)^. Some symptoms, such as muscle weakness, can persist longer. No chronic effects have been reported. TTX and STX act directly on voltage-activated sodium channels in nervous tissue^8^^)^. Toxin binding to the channel blocks the diffusion of sodium ions, preventing depolarization and propagation of action potentials. All of the observed toxicity is secondary to action-potential blockage. These toxins are both heat- and acid-stable under normal conditions of storage and consumption. They are not destroyed by cooking or freezing and still cause illness when exposed to the acidic environment of the stomach.

There are 184 described species of puffer fish worldwide and they occur in freshwater, estuarine, and marine environments^9^^)^. Several of these species are consumed throughout the world, particularly in the Indo-Pacific region where puffer fish hold great cultural significance. In several species, the gonads (mainly ovary), liver, intestines, and skin can contain levels of TTX sufficient to result in human intoxication. In a few select species, the meat naturally contains enough TTX to be lethal. In addition to puffer fish, TTX has also been documented in other species including gobies, trumpet shells and other gastropods, horseshoe and xanthid crabs^10^^,^^11^^,^^12^^)^. Although occasionally consumed and associated with illness in other parts of the world, none of these species are imported into the United States for human consumption.

Among the puffer fish species known to potentially contain TTX, total toxicity can vary greatly. However, toxin presence and distribution within individual tissue pools (i.e., meat, skin, liver, etc.) does appear to be consistent within a given species. As an example, Table 1 provides the popular and scientific names for 17 species of puffer fish consumed in Japan, including which parts are considered edible. In Japan, the Ministry of Health Labour and Welfare (MHLW) provides guidance and regulation for the harvesting and consumption of puffer fish^13^^)^. Under this guidance, the meat, and sometimes the skin and testicles, depending on species and catch location, is considered safe to consume when prepared by a trained expert so as not to contaminate the edible portions with toxin from other tissues. This training also requires the ability to morphologically identify puffer fish species which is sometimes difficult for closely related species with few external distinguishing characters. For example, two species of marine puffer fish, *Lagocephalus cheesemanii* (standard Japanese name kurosabafugu) and *L. spadiceus* (standard Japanese name shirosabafugu), are considered safe for consumption in Japan and are commonly used to produce dried fish fillets and fish balls in several other countries including Taiwan and Korea^14^^)^. A closely related species, *Lagocephalus lunaris* (standard Japanese name dokusabafugu), is one of the only species known to contain dangerously high levels of TTX naturally in its flesh in addition to its viscera^15^^)^. *L. lunaris* has been associated with illness in several Indo-Pacific countries where it has been used accidentally in the production of these products^16^^,^^17^^,^^18^^,^^19^^)^. This species is not considered safe for consumption in Japan. *L. lunaris* has also been associated with illness in the United States, where it has been illegally imported under false names, such as monkfish^20^^)^. The presence and distribution of food-chain derived STX in various puffer fish species is more unpredictable^3^^)^. In select species that co-occur with blooms of STX producing harmful algae, this toxin is consistently found in high concentrations in the meat, making them unsafe to consume regardless of the preparation method^21^^)^.

**Table 1.** Edible species and parts for puffer fish as determined by the Ministry of Health, Labour, and Welfare of Japan^13^^)^.

**Species**	**Standard Japanese Name**	**Edible Parts**		
		**Muscle**	**Skin**	**Testicles**
*Takifugu alboplumbeus*	Kusafugu	Yes	No	No
Takifugu flavipterus*	Komonfugu	Yes	No	No
Takifugu pardalis*	Higanfugu	Yes	No	No
*Takifugu snyderi*	Shōsaifugu	Yes	No	Yes
*Takifugu porphyreus*	Mafugu	Yes	No	Yes
*Takifugu obscurus*	Mefugu	Yes	No	Yes
*Takifugu chrysops*	Akamefugu	Yes	No	Yes
*Takifugu rubripes*	Torafugu	Yes	Yes	Yes
*Takifugu chinensis*	Karasu	Yes	Yes	Yes
*Takifugu xanthopterus*	Shimafugu	Yes	Yes	Yes
*Takifugu stictonotus*	Gomafugu	Yes	No	Yes
*Lagocephalus inermis*	Kanafugu	Yes	Yes	Yes
*Lagocephalus spadiceus*	Shirosabafugu	Yes	Yes	Yes
*Lagocephalus cheesemanii*	Kurosabafugu	Yes	Yes	Yes
*Sphoeroides pachygaster*	Yoritofugu	Yes	Yes	Yes
*Takifugu flavidus*	Sansaifugu	Yes	No	No
Takifugu vermicularis*	Nashifugu	Yes	No	Yes

* Regulations vary based on catch location

In the United States, the Food and Drug Administration (FDA) is responsible for assuring the safety of domestic and imported seafood products distributed in interstate commerce. Seafood is regulated under the U.S. Code of Federal Regulations (21 CFR Part 123 - Fish and Fishery Products) which identifies Hazard Analysis Critical Control Point (HACCP) processes for the control of specific hazards including natural toxins^22^^)^. FDA has not developed guidelines on the safe preparation of puffer fish as provided in Japan. Therefore, due to the potential presence of the poisonous and deleterious substances TTX and STX, puffer fish and all products containing puffer fish have been generally prohibited from import into the U.S. since 1980 because no adequate sampling scheme could be implemented that could assure a safe product lot since any single fish could potentially contain a lethal dose of the toxin. The only exception being a specific agreement between the FDA and the MHLW that permits the importation of Japanese puffer fish on a limited basis, typically 2–3 shipments per year between the months of September and March, provided that each entry is inspected by an appropriate official of the Japanese government and certified as safe. The agreement contains several additional conditions including: (1) species and parts are restricted to muscles, skins and testicles of *Takifugu rubripes*, also known as torafugu or tiger puffer, excised entirely of poisonous regions and, (2) processing is undertaken by a certified person (puffer fish processing specialist) in accordance with the prefectural ordinance at a facility authorized by the governor of the prefecture or the mayor of the establishing city health center. The full conditions established under this agreement can be found in the following documents: Exchange of Letters Between Japan and the U.S. Food and Drug Administration Regarding Puffer Fish - Import Conditions for Japanese Puffer Fish^23^^)^. Any other imported puffer fish or products containing puffer fish are subject to FDA Import Alert #16-20, which permits FDA field staff to detain these products without physical examination (DWPE)^24^^)^. Any firms found to be importing puffer fish under false or misleading names to avoid these import restrictions are placed on FDA Import Alert #16-04 for species misbranding^25^^)^.

Despite these efforts, several instances of illegal importation of both toxic and non-toxic products have occurred, involving a variety of species and countries of origin. Further, consumption of some of these products has resulted in both individual cases and outbreaks of PFP. Example cases are provided below. It should be noted that these cases should not be used to estimate the total occurrence of illegal importation as these shipments are often small, sometimes only a few fish, and are either not declared or intentionally mislabeled to avoid detection. No illnesses have been reported to date from *T. rubripes* imported from Japan under the conditions of the FDA/MHLW agreement.

## Imported Puffer Fish Products

### Examples of Illegal Importation of Puffer Fish Products Not Resulting in Illness.

**Fig. 1.**
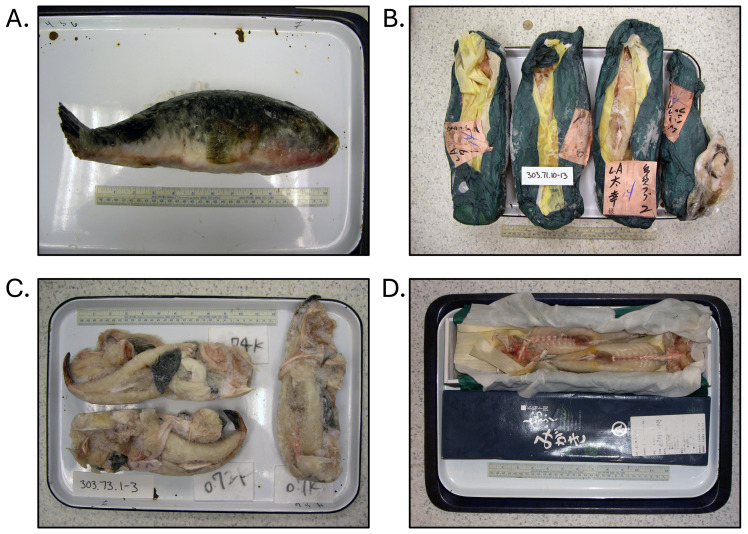
Examples of non-toxic *Takifugu rubripes* (tiger puffer / torafugu) illegally imported into the United States outside of the U.S. Food and Drug Administration / Japanese Ministry of Health, Labour, and Welfare agreement on the limited importation of puffer fish. [A], [B], and [D] were not declared at import. [C] was declared as grouper. All products were identified through DNA testing according to Handy et al.^26^^)^ and were tested for tetrodotoxin and saxitoxin by liquid-chromatography tandem mass-spectrometry according to Deeds et al.^29^^)^.

**Fig. 2.**
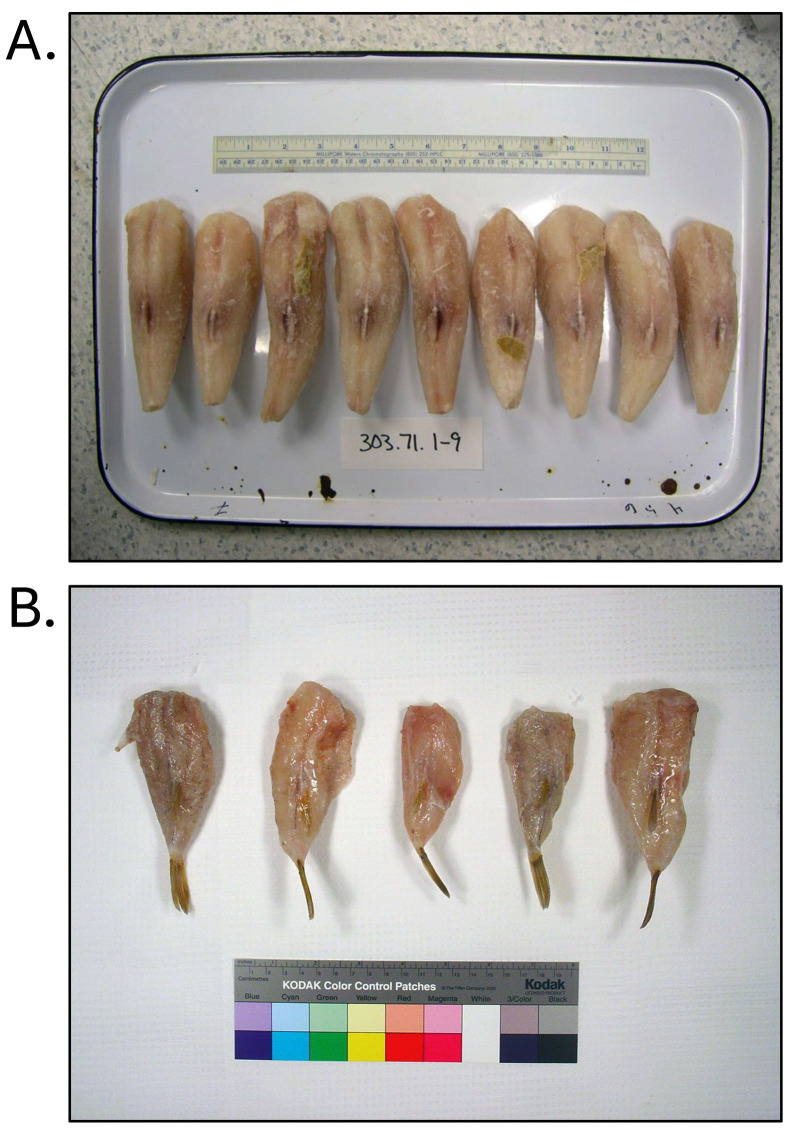
[A] Non-toxic *Lagocephalus inermis* illegally imported into the United States as filefish. [B] Non-toxic *Sphoeroides maculatus* harvested from the Mid-Atlantic region of the United States. All products were identified through DNA testing according to Handy et al.^26^^)^ and were tested for tetrodotoxin and saxitoxin by liquid-chromatography tandem mass-spectrometry according to Deeds et al.^29^^)^.

**Fig. 3.**
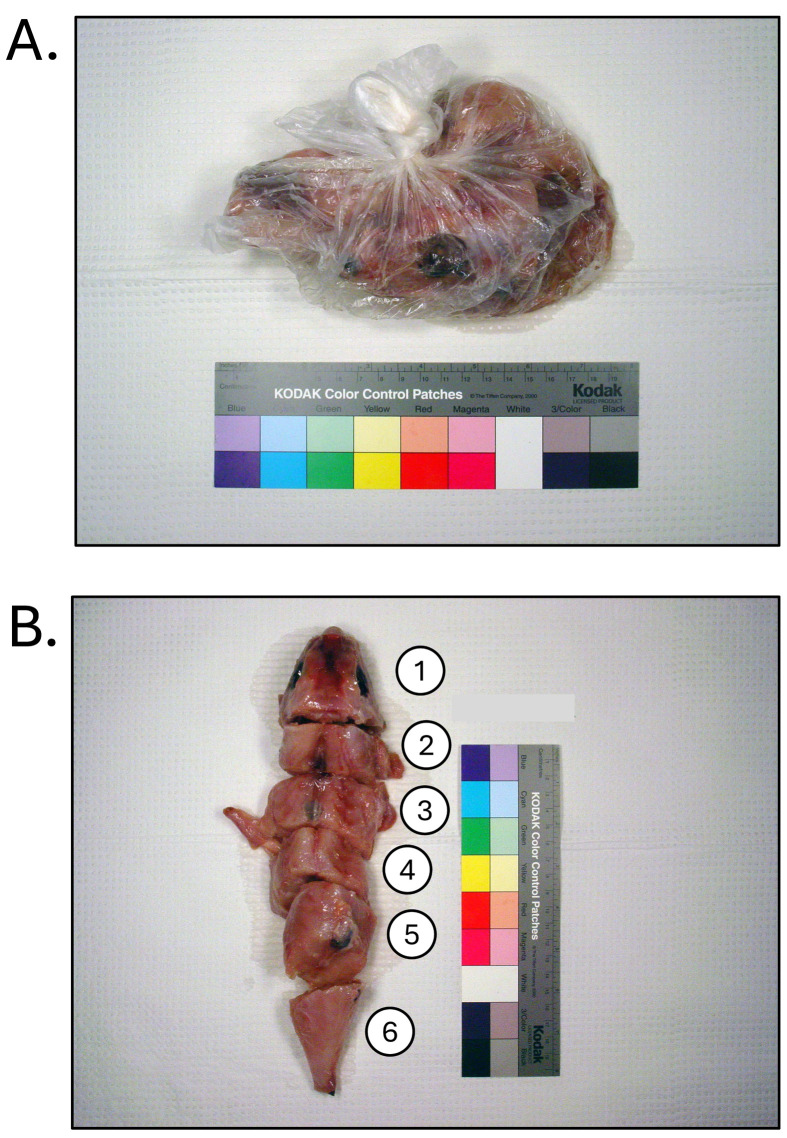
Non-toxic *Lagocephalus cheesemanii* illegally imported into the United States as brownback toadfish. [A] Product as collected. [B] Product removed from bag containing one head-on, skin-off, eviscerated fish cut into 6 pieces. Product identified through DNA testing according to Handy et al.^26^^)^ and tested for tetrodotoxin and saxitoxin by liquid-chromatography tandem mass-spectrometry according to Deeds et al.^29^^)^.

Several instances have been documented where either non-toxic/low toxicity species or toxin containing species processed according to MHLW guidelines, yet imported outside of the FDA/MHLW agreement, have been detained by FDA import inspectors because they were suspected to be puffer fish (**Table 2**). These suspicions were confirmed using DNA testing and toxin analysis^26^^,^^27^^,^^28^^,^^29^^)^. *T. rubripes*, both whole frozen and processed meat and skin, most often originated from Japan (**Fig. 1**). These products were either not declared or intentionally mislabeled (e.g., “fresh grouper”) to avoid the FDA import alert on these products. Other products made with non-toxic/low toxicity species such as *L. cheesemanii* and *L. spadiceous* (**Table 1**), were either mislabeled (e.g., as cuttlefish) or labeled improperly using other local or vernacular names such as toadfish (Figs 2,3). The FDA provides guidance to importers on the proper labeling of seafood products distributed in interstate commerce in the U.S. in the form of The Seafood List^30^^)^. This guidance prohibits the use of local or vernacular names for seafood products to avoid confusion among U.S. consumers and to assist FDA inspectors in assessing proper labeling. For example, even though toadfish is sometimes used as a vernacular name for certain puffer species in the genus *Lagocephalus*, The Seafood List restricts the use of the market name toadfish to select members of the genus *Opsanus* and *Batrachoides*. It should be noted that The Seafood List only contains market names for products allowed for sale in the United States. For puffer fish this is currently restricted to three species, *T. rubripes, Sphoeroides maculatus*, and *S. nephelus*. The illegally imported *Lagocephalus* products typically originated from countries such as China and South Korea.

**Table 2.** Examples of non-toxic puffer fish products illegally imported into the United States

**Labeled As**	**Product Form**	**DNA Result** ^1^^)^	**Tetrodotoxin Analysis** ^2^^)^
Not Declared	Whole; frozen	*Takifugu rubripes*	Not detected
Not Declared	Head-off, skin-off, eviscerated (skin on the side); frozen	*Takifugu rubripes*	Not detected
Grouper	Head-off, skin-off, eviscerated (skin on the side); frozen	*Takifugu rubripes*	Not detected
Filefish	Head-off, skin-off, eviscerated; frozen	*Lagocephalus inermis*	Not detected
Cuttlefish	Head-off, skin-off, eviscerated; dried	*Lagocephalus spadiceus*	Not detected
Brownback Toadfish	Head-on, skin-off, eviscerated; frozen	*Lagocephalus cheesemanii*	Not detected

^1^ Species determined using cytochrome c oxidase 1 mitochondrial (CO1) gene according to Handy et al.^26^^)^.

^2^ Toxin analysis based on liquid chromatography tandem mass spectrometry (LC-MS/MS) according to Deeds et al^29^^)^.

### Examples of Illegal Importation of Puffer Fish Products Resulting in Illness.

In addition to the products described above, which were typically small amounts detained at import and not causing illness, several individual cases and outbreaks of illness have been documented due to the illegal importation of puffer fish products contaminated with TTX (**Table 3**).

**Table 3.** Examples of toxic puffer fish products illegally imported into the United States and responsible for illness

**Labeled As**	**Product Form**	**DNA Result** ^1^^)^	**Tetrodotoxin Analysis** ^2^^)^
Headless Monkfish	Head-off, eviscerated, skin-on; frozen	*Lagocephalus lunaris*	10–9,600 µg/kg
Dry Fish	Head-off, eviscerated, skinned; dried	*Lagocephalus lunaris*	60–72,000 µg/kg
Not labeled	Head-on, eviscerated, skin-on; dried	*Takifugu flavipterus*	7,100 µg/kg

^1^ Species determined using cytochrome c oxidase 1 mitochondrial (CO1) gene according to Handy et al.^26^^)^

^2^ Toxin analysis based on liquid chromatography tandem mass spectrometry (LC-MS/MS) according to Deeds et al.^29^^)^

**Fig. 4.**
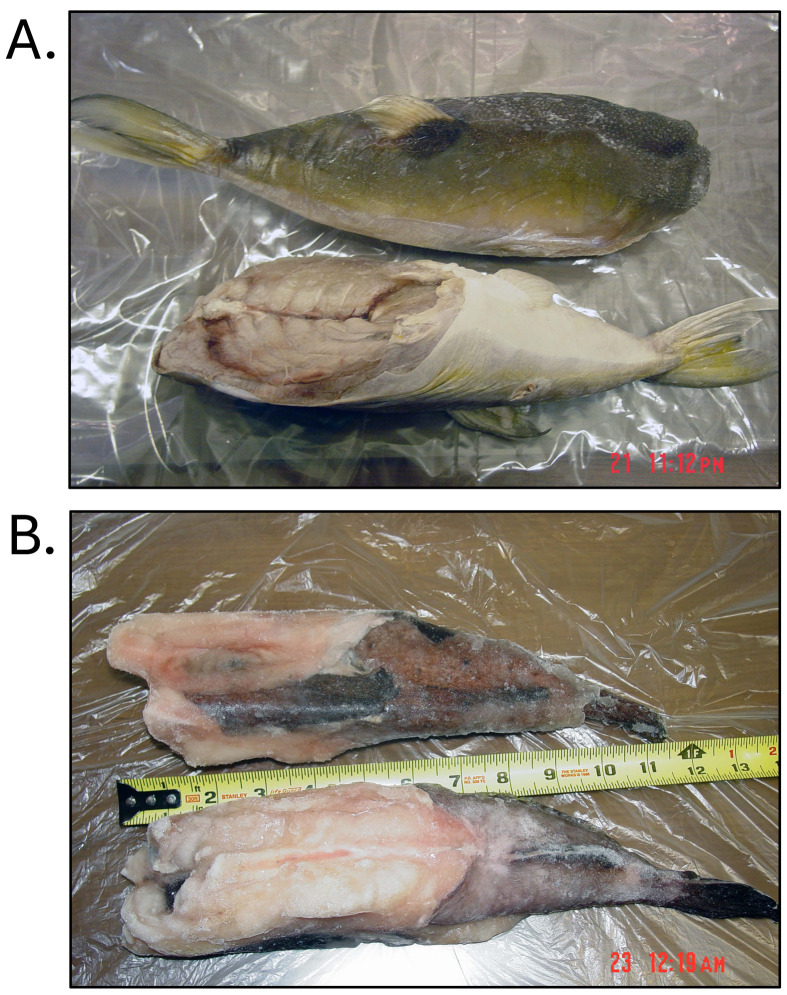
[A] Frozen, head-off, skin-on, eviscerated *Lagocephalus lunaris* found to contain 10–9,600 µg/kg tetrodotoxin, and responsible for multiple cases of puffer fish poisoning in the United States in 2007. Product imported in boxes labeled as “Frozen Monk Fish Gutted and Head-Off”. [B] Frozen, head-off, skin-on, eviscerated monkfish (*Lophius litulon*) collected at same location in boxes labeled as “Frozen Monk Fish Tail”. Products identified through DNA testing according to Handy et al.^26^^)^ and tested for tetrodotoxin and saxitoxin by liquid-chromatography tandem mass-spectrometry according to Deeds et al.^29^^)^.

In 2007, 2,800 kg of frozen, head-off, eviscerated, skin-on, *Lagocephalus lunaris* was imported into the United States from China in boxes labeled as “Headless Monk Fish”^20^^)^ (Fig. 4). This species is not considered as edible in Japan due to the potential presence of high levels of TTX in its flesh. This product was distributed to three states causing multiple illnesses. Illnesses occurred in individuals purchasing “Bok”, a Korean vernacular name for puffer fish, either in markets and prepared at home as soup, or in restaurants as “Bok Jiri” (blowfish stew) or “Bok Go Jim” (blowfish casserole). Analysis of cooked fish meat and broth from soup samples collected during this event ranged from 360–650 µg TTX/kg, while analysis of multiple uncooked fish from an unopened case collected at import found TTX concentrations in the meat ranging from 10–9,600 µg/kg^20^^)^.

**Fig. 5.**
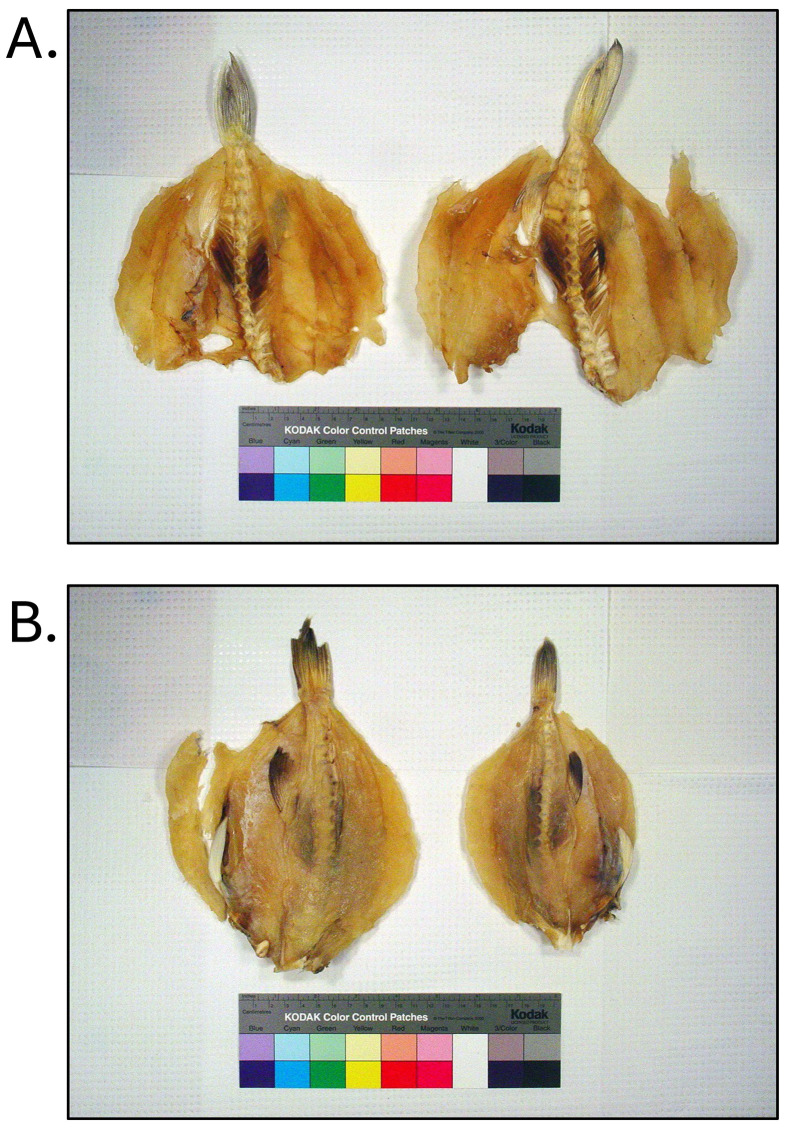
[A] Dried, head-off, skin-off, eviscerated *Lagocephalus lunaris* found to contain 60–72,000 µg/kg tetrodotoxin, and responsible for cases of puffer fish poisoning in the United States in 2014. [B] Dried, head-off, skin-off, eviscerated *Lagocephalus spadiceus* (non-toxic) illegally imported into the United States as “cuttlefish”. Products identified through DNA testing according to Handy et al.^26^^)^ and tested for tetrodotoxin and saxitoxin by liquid-chromatography tandem mass-spectrometry according to Deeds et al.^29^^)^.

**Fig. 6.**
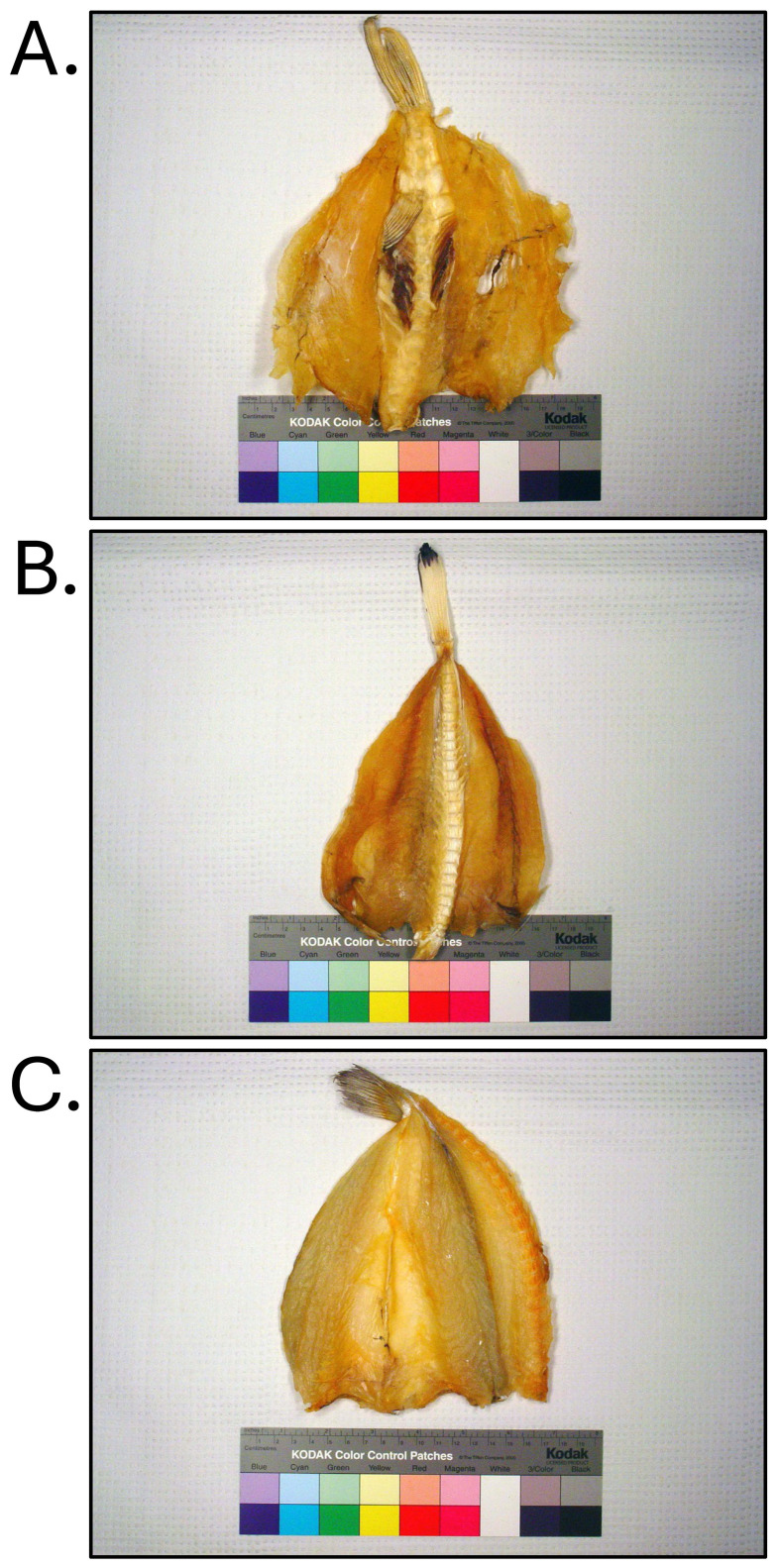
Dried, head-off, skin-off, eviscerated products collected as part of puffer fish poisoning investigation in 2015. [A] *Lagocephalus lunaris* collected at retailer and invoiced as “dry fish”. Product found to contain 850–17,000 µg/kg tetrodotoxin. [B] Similar dry fish product (non-toxic) collected at importer, identified as monkfish (*Lophius litulon*). [C] Similar dry fish product (non-toxic) collected at importer, identified as yellow croaker (*Larimichthys polyactis*). Products identified through DNA testing according to Handy et al.^26^^)^ and tested for tetrodotoxin and saxitoxin by liquid-chromatography tandem mass-spectrometry according to Deeds et al.^29^^)^.

In 2014, a small outbreak of PFP was associated with a dried, head-off, skin-off, eviscerated product identified through DNA testing as *L. lunaris*^31^^)^ (Fig. 5). The product, reported by the purchaser to be “globefish”, was acquired from an unidentified street vendor in New York City and was found to contain TTX in the range of 60–72,000 µg/kg. After similar illnesses from the same apparent product occurred in a different state in 2015, also acquired from a small market in New York City, a follow-up investigation found the same dried *L. lunaris* product, sold loose and unlabeled, containing TTX in the range of 85–17,000 µg/kg (unpublished) (Fig. 6 A). The product was sold to the market by a Chinese importer invoiced as “dry fish”. Import records for the product identified it as “sea bass”. Investigation of the importer, as well as another market that also purchased “dry fish” from the same importer, found several types of similar looking products, identified through DNA testing as monkfish (*Lophius litulon*) and yellow croaker (*Larimichthys polyactis*) (Fig. 6 B, C). No puffer fish products were found during inspection of the importer or at the other market receiving “dry fish”; therefore, the manufacturer of the dried *L. lunaris* could not be determined.

**Fig. 7.**
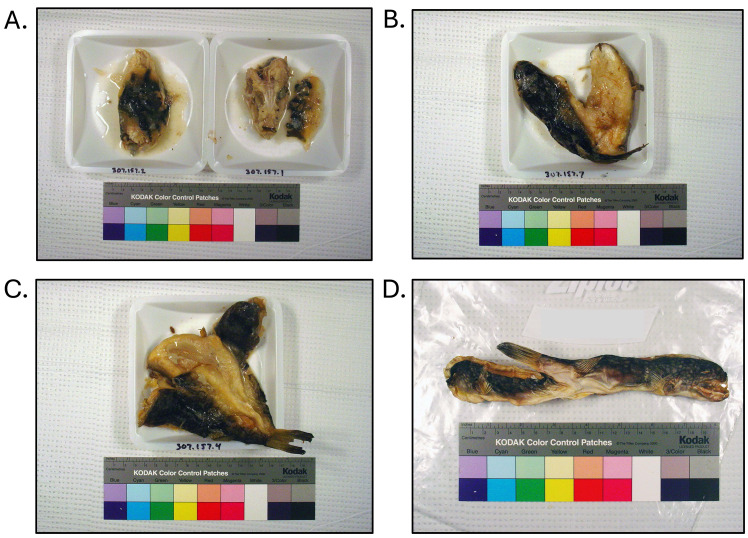
Samples collected as part of puffer fish poisoning investigation in 2014 from consumption of home prepared stew. [A] Locally caught cooked *Sphoeroides maculatus*. [B] Cooked “dry fish” product, identified as *Takifugu rubripes*. [C] Cooked “dry fish” product, identified as *Takifugu flavipterus*. [D] Uncooked, dried, head-on, skin-on, eviscerated “dry fish” product, identified as *T. flavipterus*. Stew found to contain 300 µg/kg tetrodotoxin, cooked fish found to contain 250–400 µg/kg tetrodotoxin, and product D (dried fish identified as *T. flavipterus*) found to contain 7,100 µg/kg tetrodotoxin. Products identified through DNA testing according to Handy et al.^26^^)^ and tested for tetrodotoxin and saxitoxin by liquid-chromatography tandem mass-spectrometry analysis according to Deeds et al.^29^^)^.

In 2014, a severe illness consistent with PFP was reported by a local health department in an individual who home-prepared stew using what was reported by the individual as a locally caught (domestic) puffer fish and additional “dried fish” acquired from a relative in South Korea. Samples of the stew liquid were found to contain 300 µg/kg TTX as tested by liquid-chromatography tandem mass-spectrometry (LC-MS/MS) (unpublished). Visual inspection of cooked fish recovered from the stew suggested that the additional “dried fish” were also puffer fish (Fig. 7). Multiple pieces of cooked fish were found to contain TTX ranging from 250–400 µg/kg, consistent with levels found in the stew liquid. DNA testing of cooked fish pieces recovered from the stew identified three species of puffer fish to be present: *Sphoeroides maculatus*, *Takifugu rubripes*, and *T. flavipterus* (Fig. 7 A-C). *S. maculatus* is a domestic species native to the local area and historically shown to be non-toxic^29^^)^. The cooked samples identified as *T. rubripes* and *T. flavipterus*, both non-domestic species, were an eviscerated, head-on, and skin-on product. *T. rubripes* is reported to have non-toxic meat and skin, but the skin of *T. flavipterus* (standard Japanese name komonfugu) is reported to be potentially toxic (Table 1); therefore, this species was suspected to have contaminated the stew and caused the illness. A single sample of uncooked dried fish (eviscerated, head-on, skin-on) was also obtained from the patient (Fig. 7 D). This product was determined by DNA testing to be *T. flavipterus* and found to contain 7,100 µg/kg TTX, confirming this product as the source of toxin.

## Domestic Puffer Fish

At least 19 species of puffer fish, mainly in the genus *Sphoeroides*, have been documented to occur in North America^32^^)^. In addition to regulations which require the control of hazards^22^^)^, FDA provides guidance for the control of the hazards associated with specific fish, including domestically sourced puffer fish, through the Fish and Fishery Products Hazards and Control Guidance^33^^)^. Additional state restrictions also apply in some areas.

U.S. domestic puffer fish species, mainly in the genus *Sphoeroides*, may be toxic or non-toxic. An example of a non-toxic domestic puffer fish species is *S. maculatus* (U.S. common name northern puffer). This species is typically harvested in mid-Atlantic waters (New York to Virginia) and has been marketed as “sea squab” or “chicken of the sea”^28^^)^. Restaurants have also been documented to serve this product using novel marketing names such as “sugar toads.” The closely related species *S. nephelus* (U.S. common name southern puffer), occurs in Florida and the Gulf of Mexico and has been harvested and marketed along with the *S. maculatus* in the same sea squab fishery. *S. nephelus* were also historically reported to be non-toxic, with few previous reports of illness from this fishery; however, between 2002 and 2004, 28 cases of PFP occurred along the Atlantic Coast of the U.S. and were linked to *S. nephelus* from the Indian River Lagoon system located on the east (Atlantic) coast of Florida. Analysis of meal remnants (fried meat) from an early case found STX (36,000 µg/kg measured by liquid-chromatography tandem mass-spectrometry), and no TTX^34^^)^. A follow-up study tested for both STX and TTX in three species of puffer fish native to the Atlantic coast of Florida, *S. nephalus*, *S. testudineus* (U.S. common name checkered puffer), and *S. spengleri* (U.S. common name bandtail puffer). High concentrations of STX were found in the meat of *S. nephalus* (max. 19,000 µg/kg), with little to no TTX detected, while in co-occurring *S. testudineus* and *S. spengleri*, high concentrations of TTX were detected primarily in the liver and ovaries (max. 98,000 and 28,000 µg/kg, respectively)^29^^)^. Another study analyzing for STX, only, in these same species collected throughout the state of Florida between 2002–2006, found *S. nephelus* to be the most abundant species encountered with the highest concentrations of STX occurring in fish collected from the Indian River Lagoon (max. 200,000 µg/kg in meat, as tested by enzyme linked immunosorbent assay (ELISA))^35^^)^. These studies confirmed *S. nephelus* as the source of the illnesses. It is now known that Florida puffer fish, primarily from the Indian River Lagoon system on the Atlantic coast of the state, contain STX derived from an algal source^21^^)^. Furthermore, the toxins in *S. nephelus*, from this location, occur in high concentrations in the meat, making safe preparation impossible. The State of Florida subsequently banned the commercial and recreational harvesting of all puffer species from the counties of Volusia, Brevard, Indian River, St. Lucie, and Martin, on the Atlantic coast of the state^36^^)^.

## Summary and Conclusions

Despite the possible presence of potentially lethal toxins such as TTX and STX, puffer fish hold great cultural significance and are a highly valued commodity in several Indo-Pacific countries. The Ministry of Health, Labour, and Welfare in Japan provides regulation, guidance, and training on edible species and parts that allow for the safe consumption of puffer fish in their country. In the United States, where the market for puffer fish is much smaller, the safe consumption of these products is assured through regulation and strict import restrictions, limited to only 2–3 shipments per year of a single species, excised entirely of poisonous parts by a certified specialist in accordance with Japanese regulations and the Exchange of Letters between the Japanese Government and FDA. Domestic puffer fish are regulated by FDA through the Fish and Fishery Products regulation and are restricted to non-toxic species only, as well as additional restrictions placed on their harvest by individual states. Despite these efforts, occasional cases and outbreaks of puffer fish poisoning still occur, primarily due to TTX in illegally imported, improperly prepared, or inedible species, or domestic species that have accumulated STX from the food chain.
